# Modeling reconstruction-related behavior and evaluation of influences of major information sources

**DOI:** 10.1371/journal.pone.0221561

**Published:** 2019-08-23

**Authors:** Kosuke Shirai, Nobuaki Yoshizawa, Yoshitake Takebayashi, Michio Murakami

**Affiliations:** 1 Nuclear Safety Division, Mitsubishi Research Institute, Inc., Chiyoda-ku, Tokyo, Japan; 2 Department of Health Risk Communication, Fukushima Medical University School of Medicine, Fukushima City, Fukushima, Japan; Universidad de La Frontera, CHILE

## Abstract

Reconstruction has progressed steadily since the 2011 TEPCO’s Fukushima Daiichi Nuclear Power Station accident. However, some people still hesitate to eat foods from Fukushima or to travel there, and there are concerns about the health risks of radiation. We investigated the relationships among reconstruction-related behavior, risk perception, types of information, and information sources, in order to consider appropriate measures for providing information and promoting reconstruction-related behavior a number of years after the accident. We conducted an online questionnaire survey (n = 1000) of Tokyo residents. First, a factor analysis was conducted on knowledge associated with radiation. Two factors were extracted; namely, “physical knowledge” and “health/social knowledge.” We conducted structural equation modeling to construct a model of “knowledge,” “radiation risk perception,” and “intention concerning reconstruction-related behavior.” “Intention concerning reconstruction-related behavior” decreased with “radiation risk perception” and increased with “health/social knowledge.” In addition, “health/social knowledge” negatively affected “radiation risk perception;” this effect was not large, but it was significant. Second, respondents were clarified by information sources using a cluster analysis. Clusters that included respondents who got information from public relations materials issued by municipalities and websites of administrative agencies had a higher factor score for “health/social knowledge” than other clusters. The cluster of respondents who did not get any particular knowledge had the lowest factor score, which was significant, and also had a low “perception of reconstruction.”

## Introduction

The 2011 Great East Japan Earthquake and accompanying accidents at Tokyo Electric Power Company (TEPCO)’s Fukushima Daiichi Nuclear Power Station damaged surrounding areas, especially in Fukushima Prefecture. However, reconstruction efforts have advanced steadily to date. The total gross output of Fukushima Prefecture increased in FY2015 to 108.9% of that of the pre-disaster year of 2010. In addition, the number of tourists who visited the prefecture during FY2016 recovered to 92.3% of that in the same pre-disaster year, while prefectural radiation monitoring of foods from the prefecture confirmed that the number of samples exceeding standard values decreased to 0.03% of the total during the same period [1]. Some previous studies revealed that estimated doses from foods after the accident were limited [2,3].

On the other hand, a certain number of people still avoid buying foods from Fukushima Prefecture or visiting the prefecture on group tours. Murakami et al. found dread-risk (e.g., instinctively dreaded; will increase, is difficult to reduce, has a fatal effect, is associated with genetic risk, etc.) perception of dietary radionuclides among residents living outside Fukushima prefecture was higher than among Fukushima residents [4] and Igarashi reported residents living outside Fukushima Prefecture tended to avoid purchasing foods from Fukushima compared to residents living in Fukushima in 2017 [5]. According to a 2017 survey of Tokyo residents conducted by the authors, 26.3% and 35.0% of respondents stated that “*they hesitate to eat because of concerns over radiation*” when they or their family eat foods from the prefecture, respectively. Regarding trips to Fukushima Prefecture, 28.0% and 36.9% of respondents answered that “*they hesitate to visit because of concerns over radiation*” when they or their family visit the prefecture, respectively [6]. These results indicated that anxiety over radiation was not only negatively affecting industry in the prefecture, but was also disturbing reconstruction activities. Because buying more food from Fukushima prefecture and traveling more to the prefecture would lead to the faster reconstruction of Fukushima Prefecture, the perception associated with such behavior is called “Perception concerning Reconstruction-related Behavior" hereinafter in this report.

There is also anxiety concerning the health effects of radiation caused by radioactive materials that were scattered during the accidents at TEPCO’s Fukushima Daiichi Nuclear Power Station. In the Fukushima Health Management Survey -Mental Health and Lifestyle Survey- conducted by Fukushima Medical University and Fukushima Prefecture, residents of 13 municipalities spanning evacuation order areas of the prefecture were asked about the anticipated likelihood of post-disaster radiation causing cancer and affecting the health of next-generation residents. The number of respondents who answered “likely” or “very likely” had decreased to 32.5% (causing cancer; delayed risk) and 36.1% (affecting the health of the next generation; genetic risk) in 2018 [7], and Takebayashi et al. pointed out that rates for residents living in Fukushima who feel anxiety over radiation decreased from 2012 to 2015 for risk perceptions of radiation in the Fukushima Health Management Survey [8]. Meanwhile, in our similar questionnaire survey of Tokyo residents, 53.5% (delayed risk) and 49.8% (genetic risk) of respondents answered that radiation effects would be likely or very likely [6]. There is a possibility that this recognition of the health effects of radiation may have a negative influence on “Intention concerning Reconstruction-related Behavior.” Kudo and Nakayachi examined a buying behavior model for agricultural products produced in Fukushima prefecture using structural equation modeling, which indicated that anxiety over radiation and nuclear had a negative influence on buying intention [9].

Previous studies reported that various factors affected radiation risk perception after the accident, and the governing factors of radiation risk perception included demographics, disaster-related stressors, trusted information, and radiation-related variables [8]. Meanwhile, relevance between knowledge and risk perception was inconsistent [10, 11]. It is suggested that the effects on risk perception may differ depending on types of knowledge. Miura et al., classified types of knowledge associated with radiation, namely knowledge regarding effects on the human body and scientific knowledge, and showed that the former knowledge had a closer relationship with attitude towards foods from areas around TEPCO Fukushima Daiichi Nuclear Power Station than scientific knowledge [12]. It is useful for reducing radiation risk perception and promoting reconstruction-related behavior to investigate the relationship between risk perception and types of knowledge or reconstruction-related behavior and types of knowledge. However, these relationships have not been clarified.

In addition, with respect to the risk perception of the health effects of radioactive materials, Niiyama demonstrated that image affects factors such as seriousness of health effects and perception of accumulation in the body [13], and Takenishi and Takahashi pointed out the influence of the memory of accidents on the perception of the safety of raw vegetables [14]. In this way, factors such as image and memory play important roles in risk perception. Meanwhile, in our questionnaire survey, which asked respondents how long they had caught information on the nuclear accident and revitalization in Fukushima Prefecture after the disaster, almost half answered “about two years.” [6] Based on these findings, it is highly likely that many people stopped updating relevant information after a few years and do not have the latest information. Evaluating the relation between perception of reconstruction status in Fukushima and some factors associated with reconstruction-related behavior among people living outside Fukushima may be useful for promoting behavior.

Furthermore, it is revealed that there was a relationship between risk perception and information sources [4]. It is possible that types of information source affect knowledge and influence risk perception. Clarifying the relationship between types of information source and knowledge would help in constructing a strategy for information provision measures. However, such investigations have not been reported.

This study had two objectives. First, we classified types of knowledge and found relationships between risk perception and reconstruction-related behavior. Second, we revealed a relationship between knowledge and information sources. This was the first study to find relationships among reconstruction-related behavior, risk perception, types of information, and information sources after the accident.

## Methods

### Hypothetic model of reconstruction-related behavior

**Fig 1:**
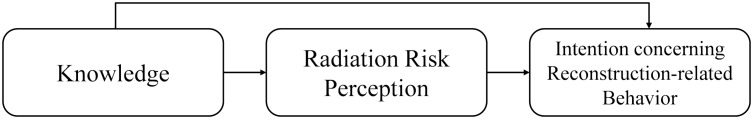
Hypothetic model of reconstruction-related behavior.

First, in order to understand the basic perception structure of reconstruction-related behavior, this study designed a simple hypothetic model consisting of three elements: “Intention concerning Reconstruction-related Behavior,” “Radiation Risk Perception,” and “Knowledge.” Here, as shown in Fig 1, we assume a one-way structure where “radiation risk perception” is influenced by “knowledge” and “Intention concerning reconstruction-related behavior” is affected by both “radiation risk perception” and “knowledge.”

In addition to the above three elements, the “perception of reconstruction status” of Fukushima Prefecture is also an important element. The perception of reconstruction status is thought to be interrelated with the above three elements, as well as limited effects on specific elements. Therefore, in this study, we also consider differences in models due to differences in perception of reconstruction status.

### Participants

Ethical approval for the study was granted by the Fukushima Medical University Ethics Committee (Ethics Committee approval number: General 29353).

The subjects of the survey were men and women in their 20s to 60s living in Tokyo and Fukushima. However, we focused on the results only from Tokyo in this study because the main purpose of this study was to learn more about risk communication in order to reduce reputational damage. Because Tokyo is located more than 200km from TEPCO’s Fukushima Daiichi Nuclear Power Station, and since most Tokyo residents were not evacuated, the perceptions of Tokyo residents may lead to reputational damage. In addition, the number of residents of Fukushima who feel anxiety concerning radiation has decreased over the last few years [8], while half of Tokyo residents felt anxiety concerning radiation [6].

The survey was conducted from August 9 to August 17, 2017, using an online questionnaire of monitors by Cross Marketing, Inc. This company is one of the largest survey companies in Japan with 4.2 million panelists. The company set up a target number of participants, grouped according to sex, age, and residential area. It asked panelists to respond to the questionnaires until this target number of respondents was collected. The target number was 100 in Tokyo from each demographic of age (20s, 30s, 40s, 50s, 60s) and sex. Elderly individuals aged 70 years or older, who may have been unfamiliar with online surveys, were excluded. Respondents provided written consent to participate in the survey before the survey was conducted.

Inappropriate responses were excluded, such as in the case of a short response time. In addition, for the purpose of understanding the general consciousness of Tokyo residents, we excluded from this survey those who were from Fukushima Prefecture or had close relatives living in the prefecture. Furthermore, those who worked for research companies or nuclear power plants and those who were in reconstruction-related posts were excluded. There were no missing data in the surveys. Respondents were encouraged to answer the survey by being awarded points that could be exchanged for Internet points. The advantages of online surveys have been described in a previous study [15]. A total of 1,000 responses in Tokyo were obtained. Table 1 shows basic information on respondents.

**Table 1:** Basic information on respondents in Tokyo.

	N(%)
Women	500(50.0%)
Men	500(50.0%)
20s	200(20.0%)
30s	200(20.0%)
40s	200(20.0%)
50s	200(20.0%)
60s	200(20.0%)
Company employees etc.	464(46.4%)
Self-employed etc.	89(8.9%)
Other	447(44.7%)
Absence of spouse	418(41.8%)
Presence of spouse	582(58.2%)
Absence of children	593(59.3%)
Presence of children	407(40.7%)

It is to be noted that the part of the data used in this study has been described in our reports [6]. In this study, we conducted another analysis using these data.

### Questionnaire

The questionnaire was made up largely of three components: questions intended to build a model for verifying the hypothesis model shown above; questions intended to evaluate differences in knowledge among information sources; and, socio-demographics.

First, the questions for model building included a set of questions concerning willingness to buy foods from Fukushima Prefecture and willingness to travel to the prefecture, in order to clarify “intention concerning reconstruction-related behavior.” Assuming that the quality and prices of foods from Fukushima Prefecture are almost the same as those from other prefectures, three options for answers were set up in connection with cases where respondents (respondents/foods) or their family members (family members/foods) would eat such foods—“eat and recommend positively (3),” “do not mind if they were from Fukushima or not (2),” and “hesitate to eat because of concerns over radiation (1).” Regarding travel to Fukushima Prefecture, similar options were set up in connection with the cases where respondents (respondents/travel) or their family members (family members/travel) would travel to Fukushima Prefecture—"travel and recommend positively (3),” “do not mind radiation (2),” and “hesitate to travel because of concerns over radiation (1).” The answers were obtained using a three-point Likert scale and scores of 3 to 1 were assigned to each affirmative answer so that those answers obtained a higher score.

Two questions were set up regarding “Radiation Risk Perception” following the questionnaire in the Fukushima Health Management Survey -Mental Health and Lifestyle Survey- conducted by Fukushima Medical University and Fukushima Prefecture [7]. Originally developed by Lindel and Barnes in the aftermath of the Three Mile Island Nuclear Pawer Station accident [16], the answers to these same questions in the Fukushima Health Management Survey were used in some studies following the accident in Fukushima [17–20]. The similarity between these indicators and dread risk perception based on Slovic’s theory [21] was discussed previously [8]. Concerning the onset of cancer and its impacts on the next and later generations, respectively, the questions asked were: “What do you think is the likelihood of damage to health (e.g. cancer onset) in later life as a result of current level of radiation exposure in Fukushima?” and “What do you think is the likelihood that the health of future (i.e. as-yet unborn) children and grandchildren will be affected as a result of current level of radiation exposure in Fukushima?” For these questions, a four-point Likert scale was used as follows: very unlikely (1), unlikely (2), likely (3), or very likely (4). It is to be noted that these questions did not represent risks to respondents, but to residents of Fukushima Prefecture. The value of Cronbach’s alpha was 0.929.

There are two types of knowledge, namely subjective knowledge [22] and objective knowledge [23]. Subjective knowledge was adopted to reduce the loads on respondents when they gave answers in this study, namely respondents chose options they knew. Two sets of questions were set up following the actual situation survey on consumer awareness of damage from rumors regularly conducted by the government [24]. First, we set up seven answer options for multiple choices, as shown in Table 2, in addition to the option “Others” and “I did not know that an inspection is being conducted.” Next, seven options were set up also for multiple choices with respect to radiation, radioactive materials, and radioactivity, as shown in the same Table 2, in addition to the options “Others” and “No particular knowledge.”

**Table 2:** Questionnaire about knowledge.

No.	A) Questions concerning the inspection of radioactive materials in food	No.	B) Questions about radiation, radioactive materials, and radioactivity
1	In cities, towns, and villages where foods exceeding the standard value are confirmed, measures are taken to prevent the same foods from being shipped, distributed, or consumed	1	Depending on the type (nuclide) of radioactive materials, there are forms of radiation such as α ray, β ray, and γ ray, which have different levels of permeability etc.
2	The inspection of foods for radioactive materials is conducted mainly in 17 prefectures in eastern Japan	2	There are two units used for radioactive substances in foods—Becquerel (Bq), representing the intensity of radiation and Sievert (Sv), representing the degree of influence on the human body
3	In accordance with the guidelines of the Nuclear Emergency Response Headquarters, local governments formulate inspection plans and conduct inspections	3	When considering the influence of radiation on the human body, it is necessary to consider the physical half-life and the biological half-life of each radioactive substance
4	The results of inspections conducted by local governments according to the inspection plan are published on the website of the Ministry of Health, Labor and Welfare	4	Receiving radiation from radioactive substances outside the human body is called “external exposure,” and receiving radiation from radioactive substances taken into the body by ingesting air, water, food, etc. is called “internal exposure"
5	In the inspection using the radioactive cesium screening method, if the inspection result exceeds the screening level (generally 1/2 (50 Bq/kg) of the reference value), a higher-precision inspection is conducted (final inspection using germanium semiconductor detector)	5	Even in our daily lives, we are subject to “external exposure” and “internal exposure” to natural radiation (exposed to radiation at a global annual average of 2.4 mSv per person from global extraterrestrial cosmic rays, radon in the atmosphere, and natural potassium 40 from in food, etc.,)
6	According to the inspection plan established by local governments, the results of inspections of pollution of agricultural land and crops are reflected	6	It is said that the risk of mortality from cancer will increase by about 0.5% if the additional dose received exceeds 100 mSv during a lifetime
7	In the examination for FY2016, 0.03% of the total number of samples exceeded the reference value	7	It is said that if the additional dose received during a lifetime is less than 100 mSv, health effects are not clarified
8	Others	8	Others
9	I did not know that an inspection is being conducted	9	No particular knowledge

Furthermore, for the purpose of examining the detailed model, as a “perception of the reconstruction status of Fukushima Prefecture,” the question set was: “*I feel that restoration and reconstruction of Fukushima prefecture are progressing*.” We used a five-point Likert scale ranging from 5 (I think so) to 1 (I do not think so).

Next, questions were designed to evaluate differences in knowledge among information sources. As sources of information concerning the reconstruction status of Fukushima Prefecture, eleven sources were suggested for multiple choices, in addition to “Other sources” and “Did not particularly obtain information:” “Websites of administrative agencies,” “Websites of universities, research institutions, and medical institution,” “Websites other than the above two sources,” “Twitter,” “Facebook and other social networking services (SNSs), excluding Twitter,” “TV and radio,” “Newspapers and magazines,” “Advertisements and leaflets,” “Public relations materials issued by municipalities,” “Circulations of regional community associations,” and “Friends and acquaintances.”

Finally, socio-demographics asked were composed of sex, age, prefecture of residence, prefecture of birth, the presence/absence of close relatives who were born or are living in Fukushima Prefecture, occupation, marital status, and presence/absence of children.

### Statistical analyses

To classify types of knowledge, we conducted a factor analysis using the maximum likelihood method and Promax rotation. Promax rotation was used because correlation between factors concerning knowledge was assumed. We extracted two factors based on a parallel analysis, scree test, and Kaiser-Guttman method. From the results of the parallel analysis, the maximum number of factors can be judged to be 2. Also, from the least squares partial correlation examined, we confirmed that the minimum factor number was 1. Based on the above results and the possibility of interpretation, the number of factors was judged to be 2.

Structural equation modeling was conducted to evaluate the model of relationships among knowledge, risk perception, and reconstruction-related behavior. For the analysis, SPSS Amos 22.0 (IBM co., USA) was used. Factor score by estimating confirmatory factor analysis was used for observed variables about knowledge and the score for each answer was used as other observed variables in the model. Four error correlations were set between observed variables constituting latent variables because it seemed that there were correlations particularly between the same behavior with different subjects (“respondents/foods” and “family members/foods,” or “respondents/travel” and “family members/travel”) and correlations between the same subjects with different behavior (“respondents/foods” and “respondents/travel,” or “family members/foods” and “family members/travel”) (S2 Fig).

Furthermore, to compare the difference in values of the path coefficient within the model due to “perception of reconstruction status” in Fukushima Prefecture, the respondents were classified into two groups—the Positive group (P group, n = 223), who responded that Fukushima is recovering, and the Not Positive group (NP group, n = 777), who responded that Fukushima is not recovering or who were unable to answer. Then, structural equation modeling was conducted again in the same model.

A cluster analysis was conducted using Ward’s method to clarify the respondents using differences among sources of information on reconstruction status in Fukushima Prefecture. When the respondents chose some options, they were assigned a score of 1 and the options not chosen were assigned a score of 0. The number of clusters was decided by the researchers based on their means of contexts.

To determine the difference in factor scores for knowledge that affected radiation risk perception and reconstruction-related behavior among clusters, an analysis of variances was conducted. F-test for equality of variance among clusters was rejected at p <. 01 and Games-Howell was used as a post-hoc test. A multiple regression analysis was also conducted using factor scores of “health/social knowledge” as an objective variable, and “physical knowledge” and the cluster divided based on information sources as an explanatory variable to adjust the factor score of “physical knowledge.” The cluster that had the largest number of respondents was set as a reference. In addition, to compare clusters concerning differences in “perception of reconstruction status,” a Chi-square test was conducted.

## Results

### Descriptive statistics for “intention concerning reconstruction-related behavior,” “radiation risk perception,” and “perception of reconstruction status”

The average score ± standard deviation (SD) was 1.89 ± 0.63 concerning food eaten by respondents (respondent/foods), compared to 1.77 ± 0.65 concerning food eaten by their family members (family members/foods). As for travel to Fukushima Prefecture, the score was 1.88 ± 0.65 for visits by respondents (respondents/travel), compared to 1.76 ± 0.66 for visits by family members (family members/travel), showing similar results to those concerning foods.

For “Radiation Risk Perception,” the average score ± SD for the onset of cancer was 2.55 ± 0.91, and the score for the influence on the next and later generations was 2.48 ± 0.90. About half of the respondents chose a higher likelihood for both items (535 respondents for the onset of illness and 498 for influence on the next and later generations).

Regarding “Perception of Reconstruction Status” in Fukushima Prefecture, the average score ± SD was 2.71 ± 1.02, indicating that not a few respondents felt that restoration and reconstruction were not progressing as fast as they had expected.

### Perception model of reconstruction-related behavior

As a result of the factor analysis, two factors were extracted as shown in Table 3. The Kaiser-Meyer-Olkin (KMO) measure of sampling adequacy was 0.917. Bartlett’s test was p < .01. Cronbach’s α in Factor 1 and Factor 2 was 0.827 and 0.784, respectively. The correlation coefficient between two factors was 0.715. The first factor was named “physical knowledge” because it contained many items indicating the physical properties of radiation. The second factor was named “health/social knowledge” because it contained many items related to food inspections currently conducted in Japan and items related to the health effects of radiation.

**Table 3:** Results of factor analysis of knowledge.

Type	Question	Selected Rate(%)	SD	Factor 1	Factor 2	Communalities
B-1	Depending on the type (nuclide) of radioactive materials, there are forms of radiation such as α ray, β ray, and γ ray, which have different levels of permeability etc	34.8	.48	**0.793**	-0.106	.520
B-2	There are two units used for radioactive substances in foods—Becquerel (Bq), representing the intensity of radiation and Sievert (Sv), representing the degree of influence on the human body	36.3	.48	**0.745**	0.000	.554
B-4	Receiving radiation from radioactive substances outside the human body is called “external exposure,” and receiving radiation from radioactive substances taken into the body by ingesting air, water, food, etc. is called “internal exposure"	39.6	.49	**0.651**	0.089	.515
B-3	When considering the influence of radiation on the human body, it is necessary to consider the physical half-life and the biological half-life of each radioactive substance	26.0	.44	**0.614**	0.112	.487
A-1	In cities, towns, and villages where foods exceeding the standard value are confirmed, measures are taken to prevent the same foods from being shipped, distributed, or consumed	47.9	.50	**0.574**	-0.107	.253
B-5	Even in our daily lives, we are subject to “external exposure” and “internal exposure” to natural radiation (exposed to radiation at a global annual average of 2.4 mSv per person from global extraterrestrial cosmic rays, radon in the atmosphere, and natural potassium 40 from in food, etc.,)	29.8	.46	**0.552**	0.141	.436
A-6	According to the inspection plan established by local governments, the results of inspection of pollution of agricultural land and crops are reflected	16.2	.37	-0.078	**0.662**	.370
A-4	The results of inspections conducted by local governments according to the inspection plan are published on the website of the Ministry of Health, Labor and Welfare	15.7	.36	-0.015	**0.630**	.384
B-7	It is said that if the additional dose received during a lifetime is less than 100 mSv, health effects are not clarified	15.0	.36	0.091	**0.565**	.401
A-5	In the inspection using the radioactive cesium screening method, if the inspection result exceeds the screening level (generally 1/2 (50 Bq/kg) of the reference value), a higher-precision inspection is conducted (final inspection using germanium semiconductor detector)	14.1	.35	-0.010	**0.544**	.288
B-6	It is said that the risk of mortality from cancer will increase by about 0.5% if the additional dose received exceeds 100 mSv during a lifetime	12.3	.33	0.121	**0.500**	.352
A-7	In the examination for FY2016, 0.03% of the total number of samples exceeded the reference value	8.6	.28	-0.097	**0.499**	.189
A-3	In accordance with the guidelines of the Nuclear Emergency Response Headquarters, local governments formulate inspection plans and conduct inspections	24.2	.43	0.106	**0.472**	.306
A-2	The inspection of radioactive substances in foods is conducted mainly in 17 prefectures in eastern Japan.	21.7	.41	0.204	**0.375**	.291
	Eigenvalue			5.3	1.3	

A) Knowledge of food inspection, B) Knowledge of radiation

**Fig 2:**
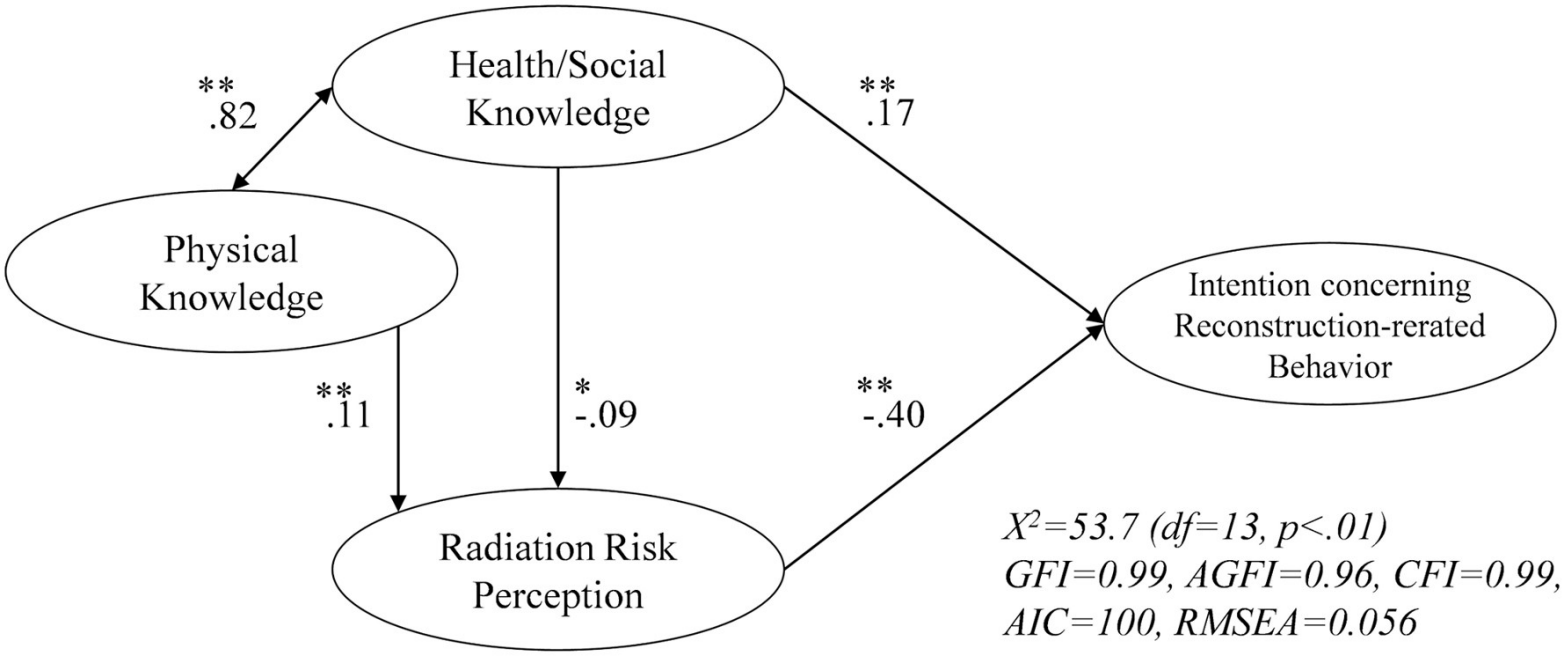
Estimated model of reconstruction-related behavior (Total). **p< .05, *p < .10.

Next, structural equation modeling was conducted to verify the model for “knowledge,” “radiation risk perception,” and “intention concerning reconstruction-related behavior.” The results are shown in Fig 2. Both paths in the figure were significant (p < .10). The values of GFI (Goodness of Fit Index), AGFI (Adjusted GFI), and CFI (Comparative Fit Index) shown in the figure are 0.95 or more, which is statistically significant. In addition, RMSEA (Root Mean Square Error of Approximation) was 0.056. From these values, it can be judged that the model is statistically acceptable [25]. The path coefficients of observed variables constituting “radiation risk perception” were 0.92 (delayed risk perception) and 0.95 (genetic risk perception). Those constituting “intention concerning reconstruction-related behavior” were 0.77 ~ 0.81. (respondents would eat foods from Fukushima; 0.78, their family members would eat them; 0.77, respondents would travel to Fukushima; 0.77, and their family members would travel there; 0.81).

In the model, first, a negative influence was seen for “radiation risk perception” on “Intention concerning reconstruction-related behavior.” “Health/social knowledge” had a positive influence on it, meaning that more “health/social knowledge” causes a stronger “Intention concerning reconstruction-related behavior.” When the values of the paths are compared, it was found that the absolute value of the path from “radiation risk perception” is higher and the influence is greater. Also, concerning the influence of knowledge on "radiation risk perception,” while the influence of “health/social knowledge” was negative and the influence of "physical knowledge" was positive, showing that whether the direction of influence is positive or negative depended on type of knowledge.

**Fig 3:**
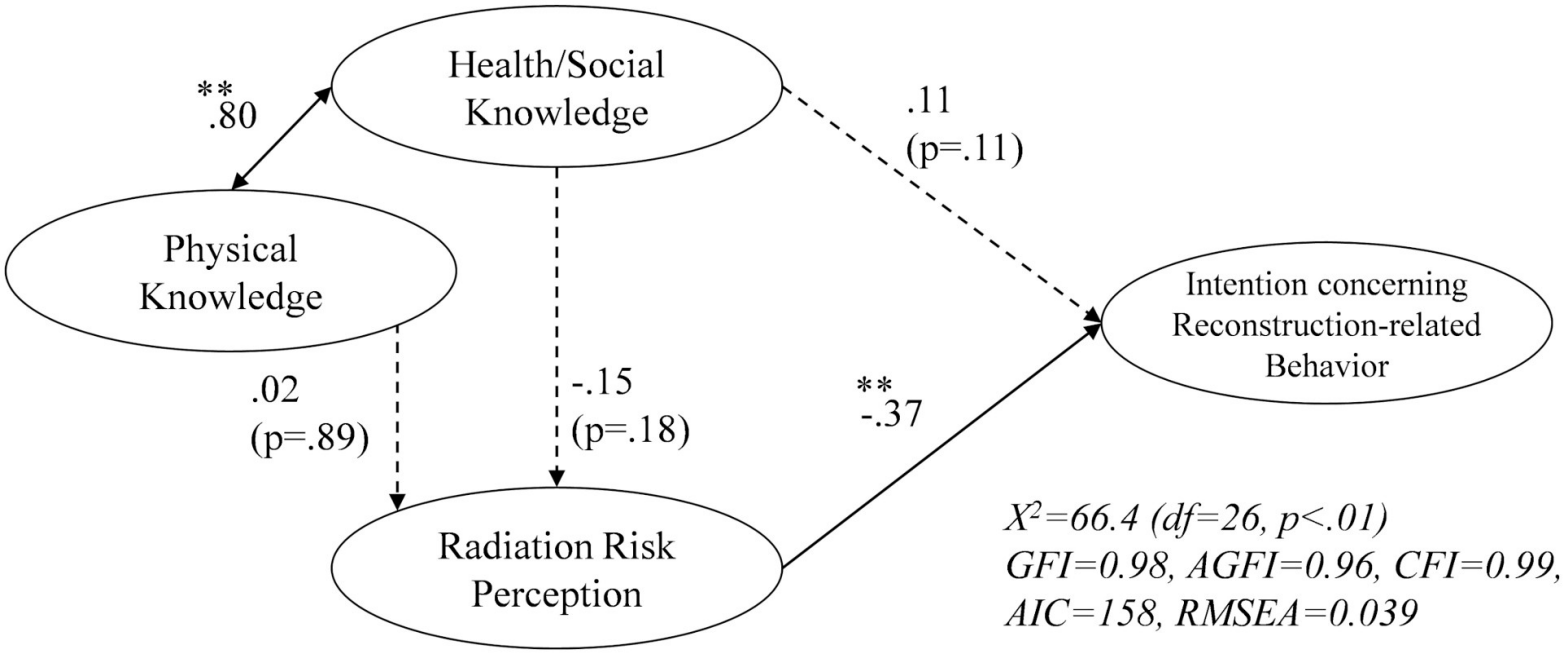
Estimated model of reconstruction-related behavior (P group). **p < .05, *p < .10. The respondents in P group (n = 223) answered that Fukushima is recovering.

**Fig 4:**
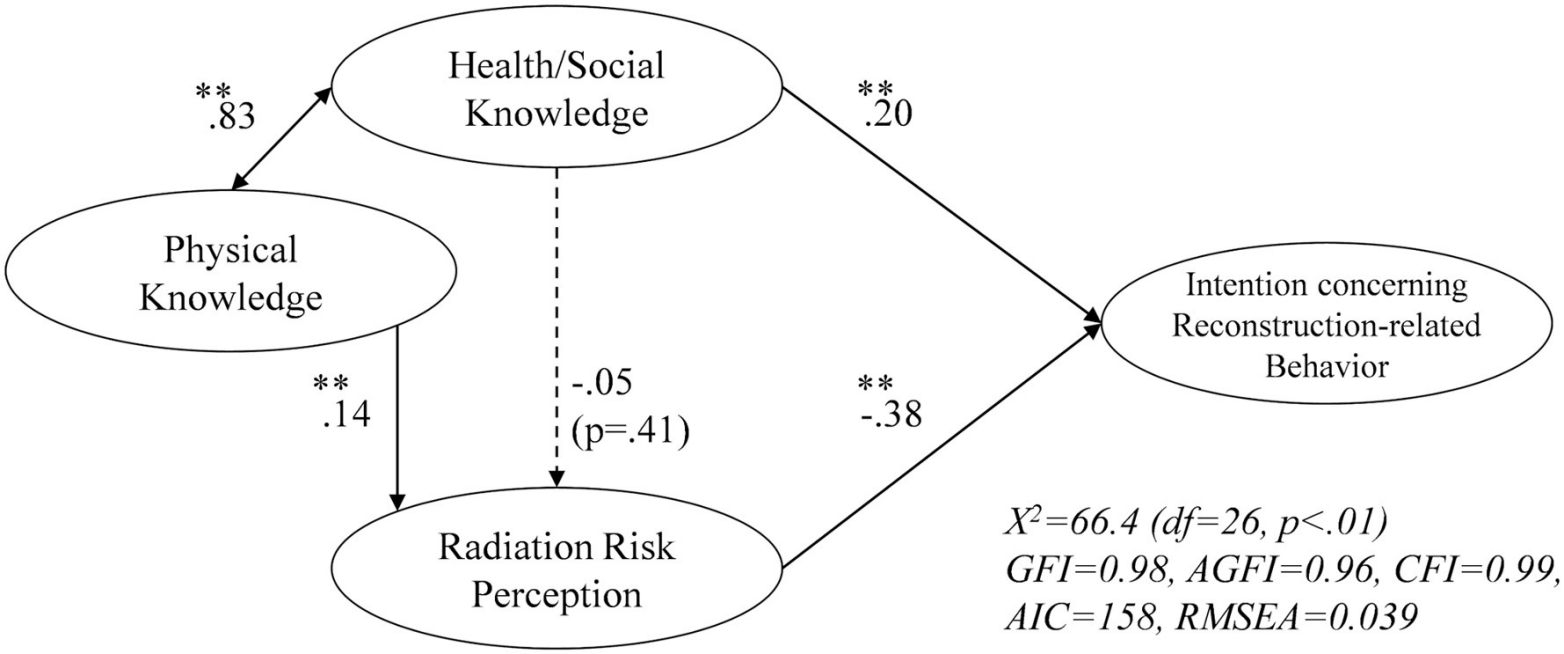
Estimated model of reconstruction-related behavior (NP group). **p < .05. The respondents in NP group (n = 777) answered that Fukushima is not recovering or who were unable to decide.

Respondents were divided into P group and NP group, and structural equation modeling was conducted again. The results for each group are shown in Figs 3 and 4. The value of RMSEA improved a little.

The coefficient of the path from “health/social knowledge” to “perception concerning reconstruction-related behavior” was moderately higher in the NP group (.20) than in the P group (.11), and a significant influence was seen only in the NP group.

The two groups were compared for the paths from the two types of knowledge to “radiation risk perception.” For the path from “health/social knowledge” to “radiation risk perception,” a negative effect was suggested in the P group, although no significant influence was seen in either group. On the other hand, for the path from “physical knowledge” to “radiation risk perception,” the path was not significant in the P group, while “radiation risk perception” significantly increased in the NP group.

In addition, a difference in path coefficient was tested statistically in the two models. No significant difference was found in either path.

### Differences among main information sources

As a result of the cluster analysis, respondents were divided into seven clusters (Table 4). This number was judged because there were too many information sources in several clusters when they were divided into fewer than seven clusters, whereas some clusters contained similar information sources when divided into more than seven clusters. Respondents were permitted to choose multiple answer options. For this reason, the number of answers does not accurately match the number of respondents in a cluster.

**Table 4:** Results of cluster analysis.

	Cluster 1	Cluster 2	Cluster 3	Cluster 4	Cluster 5	Cluster 6	Cluster 7
Websites of administrative agencies	7 (6.3%)	0 (0.0%)	48 (60.0%)	22 (12.4%)	0 (0.0%)	0 (0.0%)	15 (41.7%)
Websites of universities, research institutions, and medical institution	3 (2.7%)	0 (0.0%)	40 (50.0%)	8 (4.5%)	0 (0.0%)	0 (0.0%)	0 (0.0%)
Websites other than the above two sources	3 (2.7%)	0 (0.0%)	17 (21.3%)	5 (2.8%)	0 (0.0%)	44 (100.0%)	4 (11.1%)
Twitter	25 (22.3%)	0 (0.0%)	27 (33.8%)	0 (0.0%)	0 (0.0%)	0 (0.0%)	3 (8.3%)
Facebook and other social networking services (SNSs), excluding Twitter	31 (27.7%)	0 (0.0%)	7 (8.8%)	0 (0.0%)	0 (0.0%)	0 (0.0%)	2 (5.6%)
TV and radio	51 (45.5%)	0 (0.0%)	14 (17.5%)	178 (100.0%)	202 (100.0%)	22 (50.0%)	23 (63.9%)
Newspapers and magazines	54 (48.2%)	0 (0.0%)	6 (7.5%)	178 (100.0%)	0 (0.0%)	10 (22.7%)	22 (61.1%)
Advertisements and leaflets	21 (18.8%)	0 (0.0%)	4 (5.0%)	2 (1.1%)	0 (0.0%)	0 (0.0%)	3 (8.3%)
Public relations materials issued by municipalities	1 (0.9%)	0 (0.0%)	6 (7.5%)	0 (0.0%)	0 (0.0%)	0 (0.0%)	35 (97.2%)
Circulations of regional community associations	1 (0.9%)	0 (0.0%)	1 (1.3%)	1 (0.6%)	0 (0.0%)	0 (0.0%)	2 (5.6%)
Friends and acquantances	29 (25.9%)	0 (0.0%)	6 (7.5%)	0 (0.0%)	0 (0.0%)	0 (0.0%)	7 (19.4%)
Other sources	0 (0.0%)	0 (0.0%)	8 (10.0%)	1 (0.6%)	0 (0.0%)	0 (0.0%)	1 (2.8%)
Did not particularly obtain information	0 (0.0%)	348 (100.0%)	0 (0.0%)	0 (0.0%)	0 (0.0%)	0 (0.0%)	0 (0.0%)
Number of respondents in cluster	112	348	80	178	202	44	36
Characteristics of clusters	Various sources such as mass media, SNS, and friends	Did not particular-ly obtain informat-ion	Mainly use the website of administ-rative agencies and research institutions	Use mass media such as television, radio, and newspap-ers	Obtain relevant informat-ion only from television and radio	Mainly use websites other than govern-ment, university-ies, research institutes, etc.	Use public information on websites of administ-rative agencies or municipal-ities

**Fig 5:**
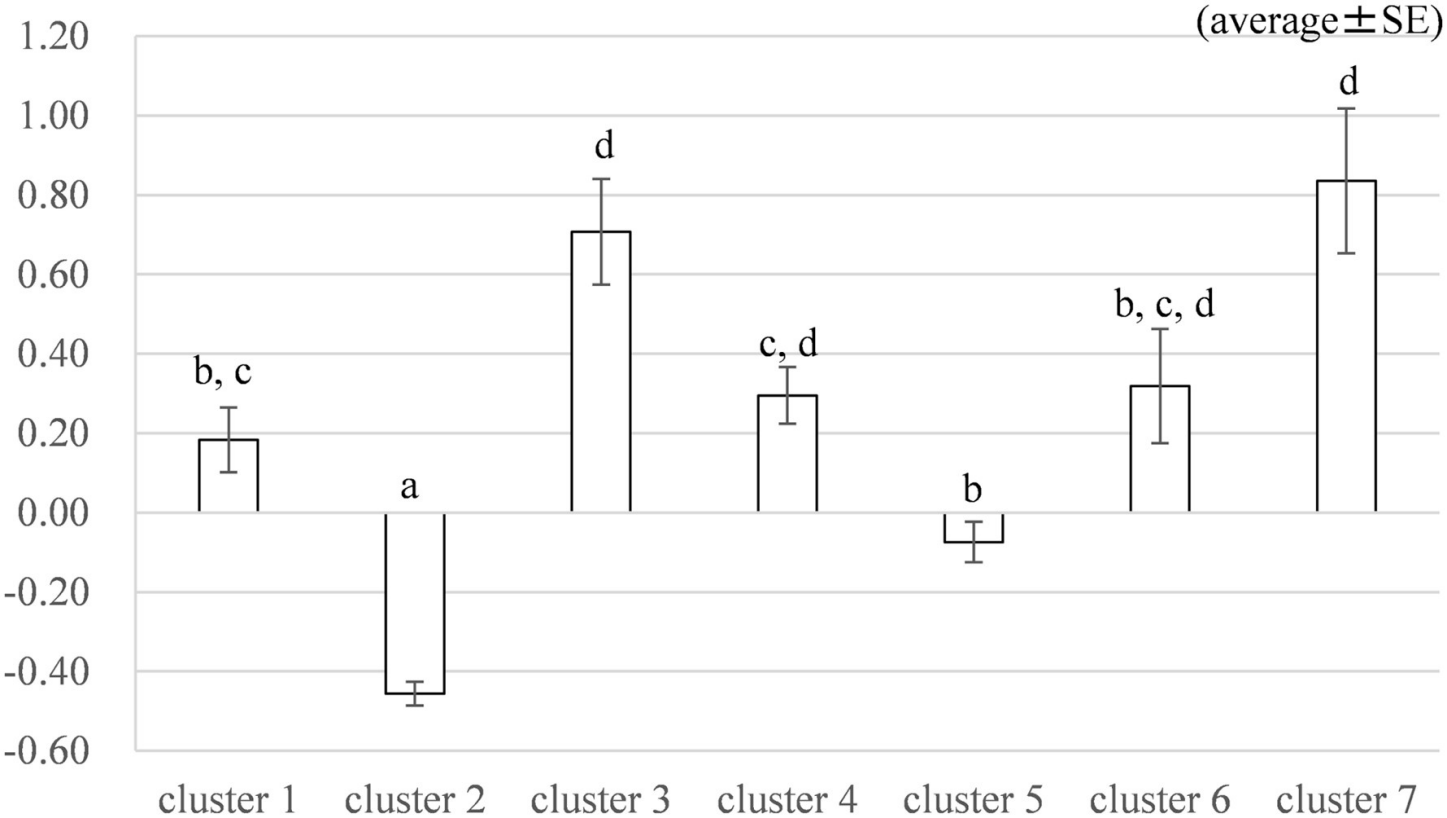
Results of analysis of variance on “health/social knowledge”. Different letters show significance.

Inter-cluster differences were compared with respect to “health/social knowledge,” which influenced “perception concerning reconstruction-related behavior” and “radiation risk perception” as discussed in the results of the SEM. As a result, the factor scores for “health social knowledge” differed significantly among the clusters (Fig 5).

Cluster 3 and Cluster 7 had a higher factor score for “health/social knowledge” than other clusters. Cluster 3 and Cluster 7 were groups that relied on public relations materials issued by municipalities and websites of administrative agencies as information sources. Cluster 2 had a significantly low factor score for “health/social knowledge,” compared to other clusters, and this cluster was a group which did not obtain any particular information. A multiple regression analysis was also conducted and the results indicate that by belonging to Cluster 3 or Cluster 7, people can increase “health/social knowledge.” These are consistent with the results above. (S9 Table).

The result of the Chi-square test showed that there was a significant difference in “perception of reconstruction” among clusters at p < .01 (Table 5). In Cluster 1, the proportion of the P group was significantly higher. This was group obtained information from various sources such as mass media, SNS, and friends. In Cluster 2, the proportion of the P group was significantly lower. This group did not obtain specific information.

**Table 5:** Results of Chi-square analysis concerning “perception of reconstruction”.

		NP group	P group
Cluster 1	Frequency	77(68.8%)	**35(31.3%)**
	Standardized residue	-1.1	**2.0**
Cluster 2	Frequency	294(84.5%)	**54(15.5%)**
	Standardized residue	1.4	**-2.7**
Cluster 3	Frequency	58(72.5%)	22(27.5%)
	Standardized residue	-0.5	1.0
Cluster 4	Frequency	136(76.4%)	42(23.6%)
	Standardized residue	-0.2	0.4
Cluster 5	Frequency	149(73.8%)	53(26.2%)
	Standardized residue	-0.6	1.2
Cluster 6	Frequency	37(84.1%)	7(15.9%)
	Standardized residue	0.5	-0.9
Cluster 7	Frequency	26(72.2%)	10(27.8%)
	Standardized residue	-0.4	0.7

χ2 = 19.3(df = 6, p < .01). Bold letters indicate p < .05.

## Discussion

### Effects of radiation risk perception and knowledge on behavior

The analysis of the perception model of behavior related to reconstruction confirmed that “perception concerning reconstruction-related behavior” was increased by lowering “radiation risk perception.” Furthermore, “health/social knowledge” has a direct and an indirect positive influence as the effect of “radiation risk perception” on “perception concerning reconstruction-related behavior.” The value of direct influence (0.17) was higher than that of indirect influence (0.04 (= -0.09×-0.40)). These results were consistent with the previous report as Kudo and Nakayachi indicated that a judgement with knowledge had positively direct and indirect (through a lowering of radiation-related anxiety) effects on intention to buy food from Fukushima [9]. The knowledge used in the judgement with knowledge was not similar to “Physical knowledge” but to “health/social knowledge.” Based on this, in order to promote reconstruction-related behavior, such as encouraging travel to Fukushima Prefecture and purchasing foods from the prefecture, it is essential to distribute relevant information on such matters as the health impacts of radiation and food inspections. It was a natural reaction to be anxious about radiation after the accident, and a high risk perception should not therefore be blamed. However, it should be also noted that a low risk perception might act to enhance reconstruction-related behavior.

The same analysis also showed that “radiation risk perception” was reduced by “health/social knowledge” and enhanced by “physical knowledge.” The relationship between risk perception and knowledge was not consistent [10,11]; however, we indicated that the effects of knowledge on risk perception depended on types of knowledge. These effects were weak, but significant.

Dose exposure at the time of the accident was limited and strict management such as restrictions on the distribution of foods has been conducted. Getting “health/social knowledge” may reduce anxiety over radiation in the circumstances, whereas “physical knowledge” seemingly increased anxiety over radiation because a possibility of effects of radiation on their health was perceived through physical knowledge about radiation. On the other hand, Wim et al. indicated that risk perception increased information needs and information seeking behavior [26]. There was a possibility of a bidirectional relationship between knowledge and risk perception; however, it is highlighted that “health/social knowledge” affected “radiation risk perception” negatively even if the possibility of bidirectional relationship was taken into account.

Factors other than knowledge were presumed to have a strong relationship with risk perception because the effects of “health/social knowledge” on “radiation risk perception” were significant, but weak. In the previous study, gender, age, and evacuation experience were reported as factors [8]. In general, risk perception reflected a cultural world view [27] and was not easily changed [28]. Nevertheless, our results offered important suggestions for the possibility of intervening in risk perception with knowledge. Most of the selection rate concerning knowledge-related questions answered by those living in Tokyo was approximately 10% lower than in Fukushima. In addition, Tokyo residents had a higher risk perception than Fukushima residents [6]. Following the accident in Fukushima, various anxiety-reducing activities were initiated. For example, Fukushima Medical University offered health counseling to city residents, including those in the evacuation area [29]. It is possible that Fukushima residents acquired knowledge about radiation, thus reducing their radiation risk perception, as a result of these activities.

In addition, the results of model verification on groups having different levels of perception of reconstruction status suggested that when people believe that reconstruction is in progress, “health/social knowledge” is highly likely to reduce “radiation risk perception.” Furthermore, the path from “physical knowledge” to “radiation risk perception” was significantly positive only in the NP group. From these findings, in order to reduce radiation risk perception, it is considered important first to know the reconstruction status.

### Relationship between knowledge and information sources

The results of knowledge differences due to information sources indicated that factor scores of “health/social knowledge” were higher in Cluster 3 and Cluster 7, which relied for information on administrative websites and public relations materials issued by municipalities. This suggests that administrative agencies and local governments provide more information, or that other sources do not provide sufficient information on health effects. Murakami et al. showed trust in central government as information sources contributed negatively to radiation risk perception [4]. People who trusted central government might have a low radiation risk perception by getting information about “health/social knowledge” from the central government. Regarding the reconstruction status of Fukushima Prefecture, many respondents in the group not obtaining information on reconstruction status did not think that reconstruction was proceeding. This indicated that a certain number of people only have information that was initially provided immediately after the accidents at TEPCO’s Fukushima Daiichi Nuclear Power Station.

In conclusion, to raise “perception concerning reconstruction-related behavior,” it is necessary to increase “health/social knowledge” and to reduce “radiation risk perception.” Also, because “radiation risk perception” is reduced by “health/social knowledge,” it is important to obtain “health/social knowledge” both directly and indirectly. In addition, it was found that groups who use information from administrative agencies were likely to have “health/social knowledge.” From this, two approaches are suggested to enhance “health/social knowledge.” First, more opportunities should be provided for many people to access administrative information. It may be useful for administrative agencies to have several channels with various media and people can reach administrative information indirectly. Second, more information related to “health/social knowledge” should be disseminated by other media. Little information on health effects attributed to radiation was contained in TV programs a month after the accident [30]. It seemed to have a big influence that TV, newspapers, and magazines used by many people as information sources gave “health/social knowledge.”

In order to reduce “radiation risk perception,” that is, to eliminate misunderstandings concerning the health impacts of radiation, post-accident information should be constantly updated as well as knowledge on the progress of reconstruction activities, rather than conveying basic physical information on radiation. In addition, it would be effective to convey social knowledge, such as information on the health effects of radiation and food inspections. Communicating only physical basic knowledge of radiation without health/social knowledge to people who do not think that reconstruction is progressing may increase radiation risk perception. In particular, Cluster 2 (did not particularly obtain information) showed the lowest health/social knowledge and perception of reconstruction. This cluster has the largest number of people and a third of the people in all clusters. In order to eliminate misunderstandings of the health effects of radiation, it would be particularly important to update information for people who have no particular information on the reconstruction status of Fukushima Prefecture.

### Limitations of this study and future perspectives

This study had some limitations. The first limitation is a potential participant bias. We used an online questionnaire survey, which may introduce biases. However, participants with no interest in the topic may have been motivated to respond to the questionnaire by receiving reward points. This may be more effective for reducing biases than mail surveys or central location testing. In addition, there were insignificant differences in risk perception of nuclear power plants between interview and online survey [31].

Second, we conducted the survey more than six years after the accident, and this was not a crisis, but a post-crisis situation, namely recovery situation. There is room for consideration as to whether our findings can be used for risk communication in a crisis situation.

Third, this was a cross-sectional study and causality was not necessarily clarified. In order to determine it, a cohort study or an intervention study needs to be conducted. After the accident, content about radiation was taught at some high schools and junior high schools proactively, and attention should be paid to its effects.

Despite these limitations, we found that the relationship between knowledge and risk perception varied among types of knowledge and that “health/social knowledge” might be effective for promoting “perception concerning reconstruction-related behavior” and for lowering “radiation risk perception.” In addition, people who used administrative agencies as information sources were likely to have much information about “health/social knowledge.” These findings would be particularly useful for risk communication in a recovery situation.

## Supporting information

S1 Fig Parallel analysis and eigenvalues of factor analysis. (PDF)

S2 Fig Estimated model. (PDF)

S1 Table Data of each participant. (XLSX)

S2 Table Pearson’s correlation coefficient between options of knowledge. (PDF)

S3 Table Arithmetic mean and standard deviation of observed variables (Total). (PDF)

S4 Table Pearson’s correlation coefficient between observed variables (Total). (PDF)

S5 Table Arithmetic mean and standard deviation of observed variables (P group). (PDF)

S6 Table Pearson’s correlation coefficient between observed variables (P group). (PDF)

S7 Table Arithmetic mean and standard deviation of observed variables (NP group). (PDF)

S8 Table Pearson’s correlation coefficient between observed variables (NP group). (PDF)

S9 Table Multiple regression analysis regarding “health/social knowledge”. (PDF)
