# Supplementary figures and images for: Modeling reconstruction-related behavior and evaluation of influences of major information sources
Source: PLoS One. 2019 Aug 23;14(8):e0221561. doi: 10.1371/journal.pone.0221561 (PMC6707550; doi:10.1371/journal.pone.0221561)

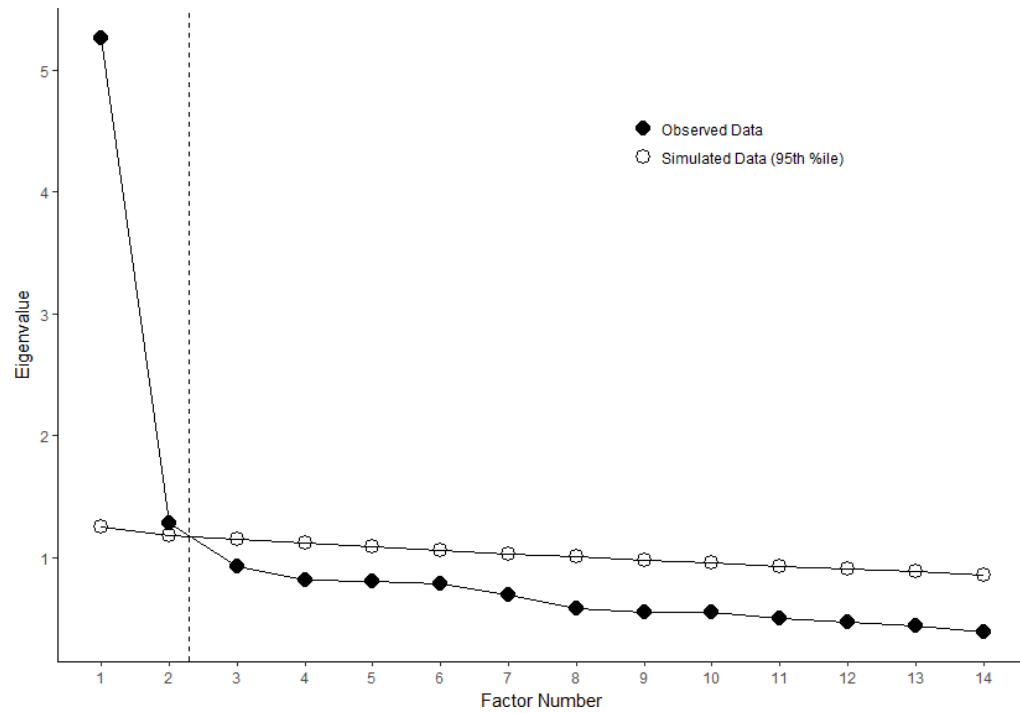

**S1 Fig. Parallel analysis and eigenvalues of factor analysis.**

Supplement: S1 Fig — (PDF) [file pone.0221561.s001.pdf]

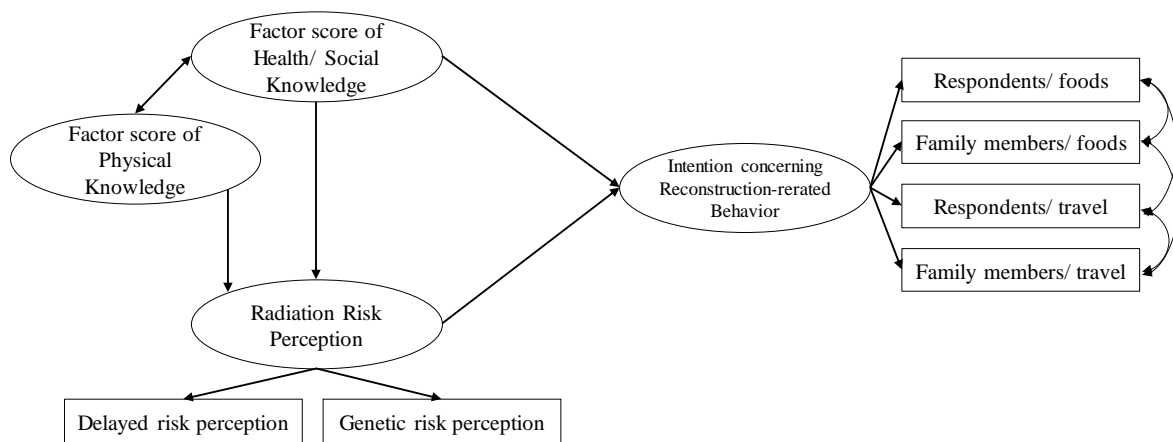

**S2 Fig. Estimated model.**

Supplement: S2 Fig — (PDF) [file pone.0221561.s002.pdf]
