# Supplementary material for: Modeling reconstruction-related behavior and evaluation of influences of major information sources
Source: PLoS One. 2019 Aug 23;14(8):e0221561. doi: 10.1371/journal.pone.0221561 (PMC6707550; doi:10.1371/journal.pone.0221561)
Supplement: S2 Table — (PDF) [file pone.0221561.s004.pdf]

## S2 table. Pearson's correlation coefficient between options of knowledge.

| | A-1 | A-2 | A-3 | A-4 | A-5 | A-6 | A-7 | B-1 | B-2 | B-3 | B-4 | B-5 | B-6 | B-7 |
|---|---|---|---|---|---|---|---|---|---|---|---|---|---|---|
| A-1 | 1.00 | 0.25** | 0.23** | 0.19** | 0.18** | 0.19** | 0.08** | 0.44** | 0.36** | 0.24** | 0.36** | 0.30** | 0.19** | 0.21** |
| A-2 | | 1.00 | 0.35** | 0.35** | 0.32** | 0.28** | 0.23** | 0.33** | 0.38** | 0.36** | 0.29** | 0.31** | 0.30** | 0.30** |
| A-3 | | | 1.00 | 0.39** | 0.26** | 0.41** | 0.16** | 0.28** | 0.36** | 0.33** | 0.34** | 0.28** | 0.27** | 0.31** |
| A-4 | | | | 1.00 | 0.36** | 0.38** | 0.28** | 0.26** | 0.33** | 0.37** | 0.34** | 0.33** | 0.31** | 0.34** |
| A-5 | | | | | 1.00 | 0.30** | 0.22** | 0.24** | 0.29** | 0.27** | 0.29** | 0.30** | 0.38** | 0.29** |
| A-6 | | | | | | 1.00 | 0.26** | 0.27** | 0.28** | 0.29** | 0.33** | 0.28** | 0.32** | 0.39** |
| A-7 | | | | | | | 1.00 | 0.19** | 0.18** | 0.18** | 0.20** | 0.25** | 0.22** | 0.32** |
| B-1 | | | | | | | | 1.00 | 0.54** | 0.51** | 0.46** | 0.44** | 0.33** | 0.33** |
| B-2 | | | | | | | | | 1.00 | 0.52** | 0.55** | 0.46** | 0.36** | 0.36** |
| B-3 | | | | | | | | | | 1.00 | 0.52** | 0.49** | 0.36** | 0.37** |
| B-4 | | | | | | | | | | | 1.00 | 0.52** | 0.34** | 0.39** |
| B-5 | | | | | | | | | | | | 1.00 | 0.36** | 0.37** |
| B-6 | | | | | | | | | | | | | 1.00 | 0.48** |
| B-7 | | | | | | | | | | | | | | 1.00 |

**p<.01
