# Supplementary material for: Modeling reconstruction-related behavior and evaluation of influences of major information sources
Source: PLoS One. 2019 Aug 23;14(8):e0221561. doi: 10.1371/journal.pone.0221561 (PMC6707550; doi:10.1371/journal.pone.0221561)
Supplement: S3 Table — (PDF) [file pone.0221561.s005.pdf]

## S3 table. Arithmetic mean and standard deviation of observed variables (Total).

| | | Arithmetic Mean | Standard Deviation |
|---|---|---|---|
| Intention concerning Reconstruction-related Behavior | Respondents/ foods | 1.89 | 0.63 |
| | Family members/ foods | 1.77 | 0.65 |
| | Respondents/ travel | 1.88 | 0.65 |
| | Family members/ travel | 1.76 | 0.66 |
| Radiation Risk Perception | delayed risk | 2.55 | 0.91 |
| | genetic risk | 2.48 | 0.90 |
| Knowledge | Physical knowledge | 0.00 | 0.93 |
| | Health/ Social knowledge | 0.00 | 0.90 |
