# Supplementary material for: Modeling reconstruction-related behavior and evaluation of influences of major information sources
Source: PLoS One. 2019 Aug 23;14(8):e0221561. doi: 10.1371/journal.pone.0221561 (PMC6707550; doi:10.1371/journal.pone.0221561)
Supplement: S4 Table — (PDF) [file pone.0221561.s006.pdf]

S4 table. Pearson's correlation coefficient between observed variables (Total).

		Intention concerning Reconstruction-related Behavior				Radiation Risk Perception		Knowledge	
		Respondents/ foods	Family members/ foods	Respondents/ travel	Family members/ travel	delayed risk	genetic risk	Physical knowledge	Health/ Social knowledge
Intention concerning Reconstruction-related Behavior	Respondents/ foods	1.00	0.74**	0.72**	0.63**	-0.27**	-0.28**	0.13**	0.17**
	Family members/ foods		1.00	0.59**	0.73**	-0.30**	-0.31**	0.04	0.10**
	Respondents/ travel			1.00	0.77**	-0.26**	-0.29**	0.14**	0.17**
	Family members/ travel				1.00	-0.29**	-0.33**	0.07*	0.11**
Radiation Risk Perception	delayed risk					1.00	0.87**	0.08*	0.04
	genetic risk						1.00	0.01	-0.03
Knowledge	Physical knowledge							1.00	0.82**
	Health/ Social knowledge								1.00

**p<.01, *p<.05
