# Supplementary material for: Modeling reconstruction-related behavior and evaluation of influences of major information sources
Source: PLoS One. 2019 Aug 23;14(8):e0221561. doi: 10.1371/journal.pone.0221561 (PMC6707550; doi:10.1371/journal.pone.0221561)
Supplement: S5 Table — (PDF) [file pone.0221561.s007.pdf]

S5 table. Arithmetic mean and standard deviation of observed variables (P group).

		Arithmetic Mean	Standard Deviation
Intention concerning Reconstruction-related Behavior	Respondents/ foods	2.01	0.66
	Family members/ foods	1.90	0.70
	Respondents/ travel	2.02	0.68
	Family members/ travel	1.91	0.72
Radiation Risk Perception	delayed risk	2.24	0.90
	genetic risk	2.14	0.88
Knowledge	Physical knowledge	0.09	0.97
	Health/ Social knowledge	0.09	0.98
