# Supplementary material for: Modeling reconstruction-related behavior and evaluation of influences of major information sources
Source: PLoS One. 2019 Aug 23;14(8):e0221561. doi: 10.1371/journal.pone.0221561 (PMC6707550; doi:10.1371/journal.pone.0221561)
Supplement: S6 Table — (PDF) [file pone.0221561.s008.pdf]

S6 table. Pearson's correlation coefficient between observed variables (P group).

		Intention concerning Reconstruction-related Behavior				Radiation Risk Perception		Knowledge	
		Respondents/ foods	Family members/ foods	Respondents/ travel	Family members/ travel	delayed risk	genetic risk	Physical knowledge	Health/ Social knowledge
Intention concerning Reconstruction-related Behavior	Respondents/ foods	1.00	0.77**	0.71**	0.69**	-0.25**	-0.28**	0.17**	0.14**
	Family members/ foods		1.00	0.62**	0.73**	-0.33**	-0.34**	0.08	0.11
	Respondents/ travel			1.00	0.82**	-0.25**	-0.29**	0.15*	0.17*
	Family members/ travel				1.00	-0.28**	-0.34**	0.15*	0.15*
Radiation Risk Perception	delayed risk					1.00	0.82**	0.01	-0.06
	genetic risk						1.00	-0.10	-0.14*
Knowledge	Physical knowledge							1.00	0.80**
	Health/ Social knowledge								1.00

**p<.01, *p<.05
