# Supplementary material for: Modeling reconstruction-related behavior and evaluation of influences of major information sources
Source: PLoS One. 2019 Aug 23;14(8):e0221561. doi: 10.1371/journal.pone.0221561 (PMC6707550; doi:10.1371/journal.pone.0221561)
Supplement: S7 Table — (PDF) [file pone.0221561.s009.pdf]

S7 table. Arithmetic mean and standard deviation of observed variables (NP group).

		Arithmetic Mean	Standard Deviation
Intention concerning Reconstruction-related Behavior	Respondents/ foods	1.85	0.62
	Family members/ foods	1.73	0.62
	Respondents/ travel	1.84	0.64
	Family members/ travel	1.72	0.64
Radiation Risk Perception	delayed risk	2.64	0.89
	genetic risk	2.58	0.88
Knowledge	Physical knowledge	-0.03	0.91
	Health/ Social knowledge	-0.03	0.88
