# Supplementary material for: Modeling reconstruction-related behavior and evaluation of influences of major information sources
Source: PLoS One. 2019 Aug 23;14(8):e0221561. doi: 10.1371/journal.pone.0221561 (PMC6707550; doi:10.1371/journal.pone.0221561)
Supplement: S8 Table — (PDF) [file pone.0221561.s010.pdf]

S8 table. Pearson's correlation coefficient between observed variables (NP group).

		Intention concerning Reconstruction-related Behavior				Radiation Risk Perception		Knowledge	
		Respondents/ foods	Family members/ foods	Respondents/ travel	Family members/ travel	delayed risk	genetic risk	Physical knowledge	Health/ Social knowledge
Intention concerning Reconstruction-related Behavior	Respondents/ foods	1.00	0.73**	0.73**	0.60**	-0.26**	-0.26**	0.11**	0.17**
	Family members/ foods		1.00	0.58**	0.73**	-0.27**	-0.28**	0.02	0.08*
	Respondents/ travel			1.00	0.75**	-0.24**	-0.27**	0.14**	0.16**
	Family members/ travel				1.00	-0.27**	-0.30**	0.04	0.09*
Radiation Risk Perception	delayed risk					1.00	0.88**	0.12**	0.08*
	genetic risk						1.00	0.05	0.03
Knowledge	Physical knowledge							1.00	0.83**
	Health/ Social knowledge								1.00

**p<.01, *p<.05
