# Supplementary material for: Modeling reconstruction-related behavior and evaluation of influences of major information sources
Source: PLoS One. 2019 Aug 23;14(8):e0221561. doi: 10.1371/journal.pone.0221561 (PMC6707550; doi:10.1371/journal.pone.0221561)
Supplement: S9 Table — (PDF) [file pone.0221561.s011.pdf]

S9 table. Multiple regression analysis regarding "health/social knowledge".

	Standardized β	p-value	VIF
Physical knowledge	0.795	0.00	1.31
Cluster 1	-0.001	0.98	1.28
Cluster 3	0.089	0.00	1.27
Cluster 4	-0.001	0.95	1.45
Cluster 5	-0.011	0.61	1.33
Cluster 6	0.014	0.46	1.13
Cluster 7	0.042	0.03	1.17
Adj-R2	0.68		
